# Supplementary material for: Ultrafast orbital tomography of a pentacene film using time-resolved momentum microscopy at a FEL
Source: Nat Commun. 2022 May 18;13:2741. doi: 10.1038/s41467-022-30404-6 (PMC9117673; doi:10.1038/s41467-022-30404-6)
Supplement: Supplementary file 1 — Supplementary Information [file 41467_2022_30404_MOESM1_ESM.pdf]

# *Supplementary Information*
# Ultrafast orbital tomography of a pentacene film using time-resolved momentum microscopy at a FEL

**K. Baumgärtner[1,2], M. Reuner[3], C. Metzger[1,2], D. Kutnyakhov[4], M. Heber[4], F. Pressacco[4], C.H. Min[1,5], T.R.F. Peixoto[1,4], M. Reiser[6], C. Kim[6], W. Lu[6], R. Shayduk[6], M. Izquierdo[6], G. Brenner[4], F. Roth[7,8], A. Schöll[1], S. Molodtsov[6,7], W. Wurth†[4,9,10], F. Reinert[1,2], A. Madsen[6], D. Popova-Gorelova[3,10], and M. Scholz[4,6*]**

[1]Experimentelle Physik 7, Julius-Maximilians-Universität, Am Hubland, 97074 Würzburg, Germany
[2]Würzburg-Dresden Cluster of Excellence ct.qmat, Julius-Maximilians-Universität, Am Hubland, 97074 Würzburg, Germany
[3]I. Institute for Theoretical Physics and Centre for Free-Electron Laser Science, Universität Hamburg, Notkestraße 9, 22607 Hamburg, Germany
[4]Deutsches Elektronen-Synchrotron DESY, Notkestraße 85, 22607 Hamburg, Germany
[5]Institut für Experimentelle und Angewandte Physik, Christian-Albrechts-Universität zu Kiel, 24098 Kiel, Germany
[6]European X-Ray Free-Electron Laser Facility, Holzkoppel 4, 22869 Schenefeld, Germany
[7]Institute of Experimental Physics, TU Bergakademie Freiberg, Leipziger Straße 23, 09599 Freiberg, Germany
[8]Center for Efficient High Temperature Processes and Materials Conversion (ZeHS), Winklerstraße 5, 09599 Freiberg, Germany
[9]Institut für Experimentalphysik, Universität Hamburg, Luruper Chaussee 149, 22761 Hamburg, Germany
[10]The Hamburg Centre for Ultrafast Imaging (CUI), Luruper Chaussee 149, 22607 Hamburg, Germany
[*]To whom correspondence should be addressed; markus.scholz@desy.de.

## 1 Sample preparation and energy calibration

For every film preparation we checked the film quality and thickness by low-energy electron diffraction (LEED). The first layer of pentacene on Ag(110) exhibits a characteristic pattern that is distinguishable from the second and subsequent layers[1,2]. Fig. S1 a) shows a typical LEED pattern of a bilayer of pentacene atop Ag(110).

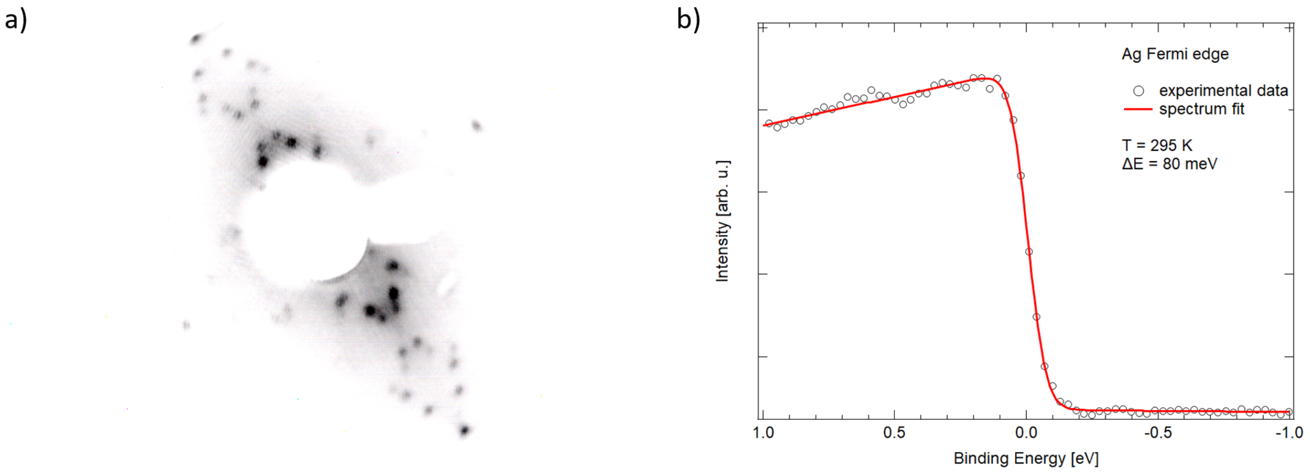


Figure S1. **Sample characterization and energy resolution.** a) LEED pattern of pentacene bilayer on Ag(110) for an electron energy of 30 eV. b) Fermi edge of an Ag(110) single crystal at 295 K. From fitting the Fermi edge we determine an energy resolution of about 80 meV.

The momentum microscope was energy calibrated at the Fermi edge of an Ag single crystal at 295 K. The instrumental energy resolution was determined by fitting the Fermi edge of Ag and considering the temperature. From the fit parameters, we estimate an energy resolution of about 80 meV.

## 2 Estimation of experimental errors

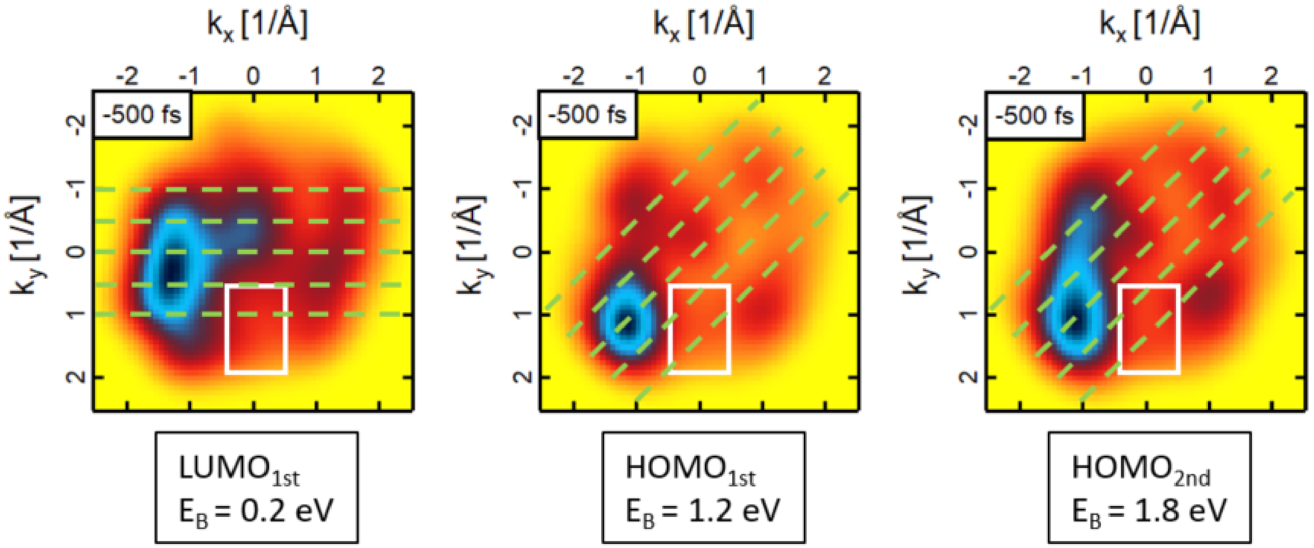


Figure S2. **Error estimation for linecuts through experimental data.** Time-resolved photoelectron momentum maps (PMMs) of pentacene of the $\mathrm{LUMO}_{1st}$ (left), $\mathrm{HOMO}_{1st}$ (middle) and $\mathrm{HOMO}_{2nd}$ (right). The green dashed lines and the white rectangular areas in the PMMs indicate, where the errors were estimated.

The 1 $\sigma$ error-bars of the momentum distribution curves are estimated by two procedures. Firstly, we determine the intensity fluctuation within a region of interest (ROI) in the PMMs. In Fig. S2 the ROI (white rectangular) is exemplary shown for $\mathrm{LUMO}_{1st}$ (left), $\mathrm{HOMO}_{1st}$ (middle) and $\mathrm{HOMO}_{2nd}$ (right) for $\tau_{\mathrm{Delay}} =$ -500 fs. The ROI does not contain any signal originating directly from the molecular orbitals, as shown by the simulations of the free molecule. We assume that the intensity is constant within the ROI and over time. Thus, we estimated an experimental error from the intensity fluctuation. Secondly, we compare the momentum distributions at two time steps before time zero ($\tau_{\mathrm{Delay}} =$ -500 fs and $\tau_{\mathrm{Delay}} =$ -150 fs). We analyze five linecuts along the direction in momentum space, marked by the green dashed lines in Fig. S2. The 1 $\sigma$ error-bars are then calculated as the mean of both errors.

## 3 Theoretical calculations

The PMMs are calculated using the plane-wave approximation (PWA) to the photoelectron wave function, which has been shown to be appropriate for the calculation of angle-resolved photoemission spectra from $\pi$ orbitals of planar molecules consisting of light atoms at small angles between the polarization of the probe pulse and momentum of a photoelectron[3–5]. Within the PWA, the photoelectron probability depending on the photoelectron momentum $\mathbf{q}$ can be directly connected to the Fourier transform of the Dyson orbital[3]

$$P(\mathbf{q}) \propto |\boldsymbol{\varepsilon}_{\mathrm{in}} \cdot \mathbf{q}|^2 \sum_{F,\sigma} \delta_{\varepsilon_e, \omega_{\mathrm{in}} - E_F^{N_{\mathrm{el}}-1} + E_I^{N_{\mathrm{el}}}} \left| \int d^3 r e^{-i\mathbf{q}\cdot\mathbf{r}} \phi_F^D(\mathbf{r}, t_p) ) \right|^2, \tag{1}$$

where a Dyson orbital is an overlap function between the $N$-electron wave function of an initial state $\Psi_I^N$, and the $(N-1)$-electron wave function $\Psi_F^{N-1}$, produced by ionization

$$\phi_F^D(\mathbf{r}) = \sqrt{N} \int \Psi_I^N(\mathbf{r}_1, \cdots, \mathbf{r}_N) \Psi_F^{N-1}(\mathbf{r}_2, \cdots, \mathbf{r}_N) d\mathbf{r}_2 \cdots \mathbf{r}_N. \tag{2}$$

$E_I^{N_{\mathrm{el}}}$ and $E_F^{N_{\mathrm{el}}-1}$ are energies of the states $\Psi_I^N$ and $\Psi_F^{N-1}$, correspondingly. $\omega_{\mathrm{in}}$ is the photon energy and $\boldsymbol{\varepsilon}_{\mathrm{in}}$ is the direction of the polarization vector of the photoionizing pulse. The summation in Eq. 1 runs over all possible final states $F$ of the ionized system and indicates that one can detect a photoelectron with the energy $\varepsilon_e$, if the energy difference between $N$-electron and $N-1$ electron state match $\omega_{\mathrm{in}} - \varepsilon_e$.

### 3.1 Isolated pentacene

For the ground-state calculations, we assume that the Dyson orbital is the molecular orbital, from which a photoelectron was detached. In this case, we apply Koopmans theorem[6] and assume that the binding energies of electrons are the negative energies of the corresponding orbitals. These approximations are used for the calculated PMMs for isolated pentacene and pentacene on a substrate in Fig. 2 in the main text.

For the calculation of PMMs for pentacene in the excited-state, we apply the RASSCF approach[7]. The calculation of the neutral excited state $\Psi_I^N$ is converged with a total of 22 active orbitals. 11 active orbitals are doubly occupied except for a maximum of one hole (RAS1 orbitals) and 11 active orbitals are unoccupied except for a maximum of one electron (RAS3 orbitals).

The Cationic final states $\Psi_F^{N-1}$ after the ionization are computed by assuming that the RAS1 orbitals are doubly occupied except for a maximum of two holes. The Dyson orbital within the RASSCF approach is a superposition of molecular orbitals[8]. The cationic final state that led to ”HOMO$_{\mathrm{2nd}}$” PMMs in Fig. 4(i)–(k) in the main text was clearly distinguishable from other final states, and the corresponding Dyson orbital had a major contribution of a HOMO orbital.

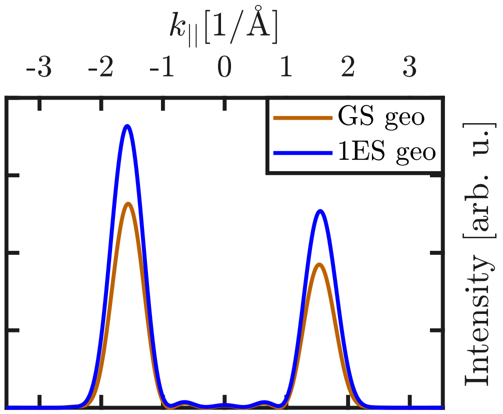


Figure S3. **Calculated momentum distribution curves for the first excited singlet state of isolated pentacene in the ground-state and excited-state geometry.** The curves correspond to the diagonal cut $k_x = -k_y$ in the PMMs of Fig. 4(m) and (n) in the main text.

Fig. S3 shows the calculated momentum distribution curves (MDCs) resulting from the first excited singlet state of isolated pentacene in the ground-state and excited-state geometry. We observe an intensity increase in the MDCs after the geometry relaxation.

### 3.2 Pentacene adsorbed on a substrate

The substrate is simulated with a cluster of silver atoms. Its geometry is based on the atomic structure of the bulk Ag that has the face-centered cubic lattice with the nearest-neighbor distance of $d = 2.942$ Å[9]. The cluster consists of 20 atoms in the first and 12 in the second layer, as depicted in Fig. S4. We use the method of pseudo-hydrogen passivation[10] to saturate the bonds of Ag atoms on the surface of the cluster that are in the bulk of the real surface. The contribution of the pseudo-hydrogens is excluded from the calculation of PMMs. We chose the relative orientation of the pentacene to the cluster as determined in Ref.[1]. Due to computational challenges, we could not determine a possible slight geometry deformation of pentacene due to the interaction with the surface[11] as well as the distance between pentacene and the surface. We used a simplified approach, in which we decreased the distance between the molecule and the cluster until the orbital of the LUMO-type character gets occupied as demonstrated in the experiment. This distance corresponded to 2.2 Å. Experimental data for the distance between pentacene and Ag(110) surface is missing. However, we can roughly estimate it with PTCDA molecule on Ag(110) surface, since PTCDA shows a similar adsorption distance on Ag(111)[12] as pentacene[13]. An experiment revealed a distance in the range of 2.3 Å up to 2.6 Å depending on the specific atom for PTCDA on Ag(110)[11]. Therefore, the distance of 2.2 Å between pentacene and Ag(110) is a reasonable assumption.

The electronic structure of the system is computed within the Hartree-Fock theory and only the neutral ground state is considered for calculations. We checked that the structure of the orbitals of the predominately molecular-type character and

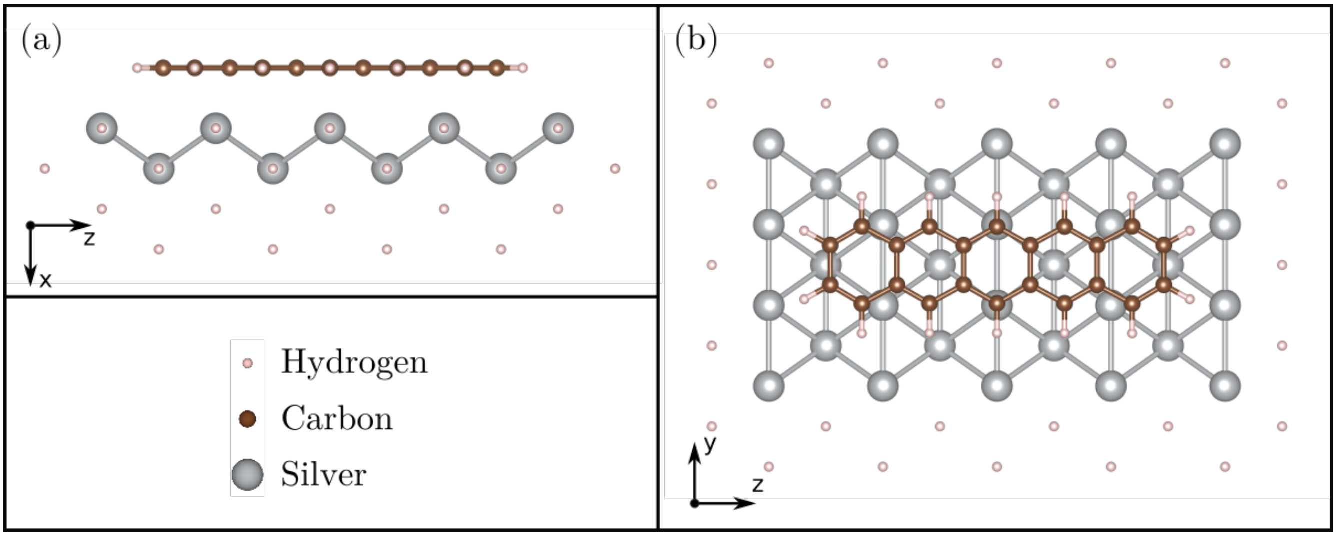


Figure S4. **Geometry of pentacene on the approximated Ag(110) cluster.** The side-view (a) and top-view (b) is visualized.

their relative energies to each other did not noticeably change with the increase of the cluster size in any direction. The eventual cluster size was selected such that it correctly represents the photoemission signal stemming from the substrate relatively to the signal of the molecule. The cluster size in the in-plane directions, i.e. $y$ and $z$, represents the unit cell size of the sample[2]. Due to the surface sensitivity of the experiment, photoelectrons mainly emitted from the two topmost silver layers account for the measured intensities, which is reproduced by our model.

The orbitals of the pentacene on the cluster system consist of orbitals of three types: ones that have only silver contribution, hybridized orbitals that have the major contribution of molecular-type orbitals and some contribution of silver orbitals (see Fig. 3 in the main text), and orbitals that have the minor contribution of molecular-type orbitals and the major contribution of silver orbitals. Orbitals of all types contributed to PMMs in Fig. 2(k)–(m) in the main text with energies within the integration region of 500 meV around the energies of the molecular-types orbitals.

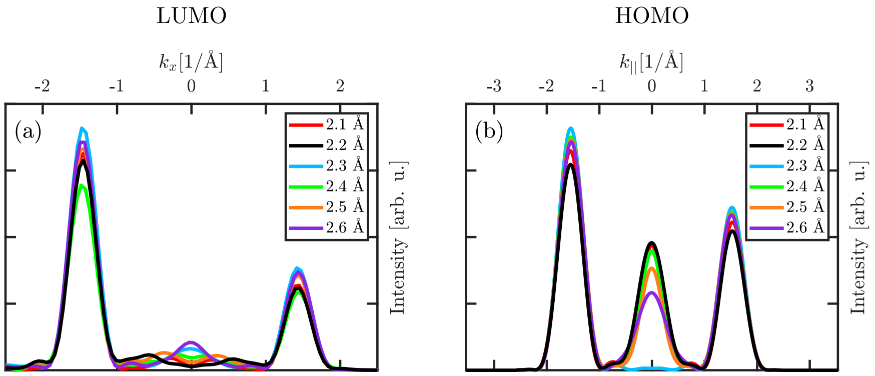


Figure S5. **Calculated momentum distribution curves.** Momentum distribution curves for the (a) LUMO-type and (b) HOMO-type orbitals depending on the distance between pentacene and substrate. The direction of the curves is indicated by the dashed lines in the corresponding PMMs in Fig. 4 in the main text. Note that the ratio of the intensity of the left peak to the intensity of the right peak is different from the one in the experimental figures, because we do not take the tilt of the molecule by 6 degrees into account for the calculation for pentacene on a cluster. The plots indicate that the intensity of the central feature of the PMMs depends on the distance between pentacene and the substrate.

In Fig. S5, we show that the intensity of the central feature in the experimental PMMs depends on the distance between the molecule and the cluster. The change of the relative distance between the molecule and the substrate after the excitation is a possible explanation for the time-dependent changes in the PMMs for the first layer of pentacene in Fig. 4 in the main text.

## References


1. Wang, Y. L. *et al.* Structural evolution of pentacene on a Ag(110) surface. *Physical Review B* **69**, 075408 (2004).
2. Grimm, M. *et al.* Molecular orbital imaging beyond the first monolayer: Insights into the pentacene/Ag(110) interface. *Physical Review B* **98**, 195412 (2018).
3. Puschnig, P. *et al.* Reconstruction of molecular orbital densities from photoemission data. *Science* **326**, 702–706 (2009).
4. Puschnig, P., Koller, G., Draxl, C. & Ramsey, M. G. *Small Organic Molecules on Surfaces: Fundamentals and Applications*, 3–23 (Springer Berlin Heidelberg, Berlin, Heidelberg, 2013).
5. Puschnig, P. & Lüftner, D. Simulation of angle-resolved photoemission spectra by approximating the final state by a plane wave: From graphene to polycyclic aromatic hydrocarbon molecules. *Journal Electron Spectroscopy Related Phenomena* **200**, 193–208 (2015).
6. Koopmans, T. Über die Zuordnung von Wellenfunktionen und Eigenwerten zu den Einzelnen Elektronen Eines Atoms. *Physica* **1**, 104–113 (1934).
7. Malmqvist, P. A., Rendell, A. & Roos, B. O. The restricted active space self-consistent-field method, implemented with a split graph unitary group approach. *The Journal Physical Chemistry* **94**, 5477–5482 (1990).
8. Popova-Gorelova, D., Küpper, J. & Santra, R. Imaging electron dynamics with time- and angle-resolved photoelectron spectroscopy. *Phys. Rev. A* **94**, 013412 (2016).
9. Hume-Rothery, W. & Reynolds, P. A high-temperature debye-scherrer camera, and its application to the study of the lattice spacing of silver. *Proceedings Royal Society London, Series A: Mathematical Physical Sciences (76,1906-)* **167**, 25–34 (1938).
10. Evarestov, R. *Quantum Chemistry of Solids: LCAO Treatment of Crystals and Nanostructures*. Springer Series in Solid-State Sciences (Springer Berlin Heidelberg, 2013).
11. Mercurio, G. *et al.* Adsorption height determination of nonequivalent c and o species of ptcda on ag(110) using x-ray standing waves. *Phys. Rev. B* **87**, 045421 (2013).
12. Hauschild, A. *et al.* Molecular distortions and chemical bonding of a large $\pi$-conjugated molecule on a metal surface. *Phys. Rev. Lett.* **94**, 036106 (2005).
13. Duhm, S. *et al.* Pentacene on Ag(111): correlation of bonding distance with intermolecular interaction and order. *ACS applied materials & interfaces* **5**, 9377–9381 (2013).
